# Genetic Confirmation of Mungbean (*Vigna radiata*) and Mashbean (*Vigna mungo*) Interspecific Recombinants using Molecular Markers

**DOI:** 10.3389/fpls.2015.01107

**Published:** 2015-12-15

**Authors:** Ghulam Abbas, Amjad Hameed, Muhammad Rizwan, Muhammad Ahsan, Muhammad J. Asghar, Nayyer Iqbal

**Affiliations:** ^1^Plant Breeding and Genetics Division, Nuclear Institute for Agriculture and BiologyFaisalabad, Pakistan; ^2^Marker Assisted Breeding Group, Nuclear Institute for Agriculture and BiologyFaisalabad, Pakistan; ^3^Department of Plant Breeding and Genetics, University of Agriculture, FaisalabadFaisalabad, Pakistan; ^4^Mungbean and Lentil Group, Nuclear Institute for Agriculture and BiologyFaisalabad, Pakistan; ^5^Pakistan Atomic Energy CommissionIslamabad, Pakistan

**Keywords:** interspecific, recombination, hybridization, molecular markers, polymorphism

## Abstract

Molecular confirmation of interspecific recombinants is essential to overcome the issues like self-pollination, environmental influence, and inadequacy of morphological characteristics during interspecific hybridization. The present study was conducted for genetic confirmation of mungbean (female) and mashbean (male) interspecific crosses using molecular markers. Initially, polymorphic random amplified polymorphic DNA (RAPD), universal rice primers (URP), and simple sequence repeats (SSR) markers differentiating parent genotypes were identified. Recombination in hybrids was confirmed using these polymorphic DNA markers. The NM 2006 × Mash 88 was most successful interspecific cross. Most of true recombinants confirmed by molecular markers were from this cross combination. SSR markers were efficient in detecting genetic variability and recombination with reference to specific chromosomes and particular loci. SSR (RIS) and RAPD identified variability dispersed throughout the genome. In conclusion, DNA based marker assisted selection (MAS) efficiently confirmed the interspecific recombinants. The results provided evidence that MAS can enhance the authenticity of selection in mungbean improvement program.

## Introduction

Mungbean (*Vigna radiata* L. Wilzeck) known as green gram and mashbean (*Vigna mungo* L. Happer) as black gram are widely cultivated pulse crops belonging to family *Fabaceae.* In cereal based cropping system, these pulses are being consumed as supplemental crops. Mungbean which is more widely cultivated as compared to mashbean has some additional properties like having more easily digestible proteins and low proportions of flatulence factors (Gosal and Bajaj, 1983) but are deficient in some essential amino acids compared to blackgram (Poehlman, 1991). The present investigation was extended to interspecific hybridization of green gram and black gram in order to improve the level of essential amino acids in green gram.

Though interspecific cross of green gram, as female, to black gram has been reported successfully but the reciprocal cross was not found successful (Sen and Ghosh, 1960; Boling and Matlock, 1961; Chowdhury et al., 1977; Singh, 1981). Identification of interspecific hybrids is an important first step for self-pollinating crops as improper emasculation may result in selfed seeds. Moreover, in most cases the identification of interspecific hybrids on the basis of morphological characteristics can be difficult in field conditions due to epistasis and environmental influence. In field, F_1_ hybrids can be identified on the basis of morphological characteristics only when one of the characteristic is intermediate between male and female parent or the plant shows high resemblance to male parent (Khajudparn et al., 2012). In certain cases, confirmation of true recombinants at the DNA level is required due to limitations of morphological characteristics alone. Molecular markers have been used for various purposes including determination of genetic relationships between individuals, construction of linkage maps, population genetics, phylogenetic studies, mapping of useful genes, and marker assisted selection/backcrosses. Several molecular marker systems, including randomly amplified polymorphic DNA (RAPD), universal rice primers (URP), simple sequence repeats (SSR), amplified fragment length polymorphism (AFLP), restriction fragment length polymorphism (RFLP), and inter-simple sequence repeats (ISSR) have been used in mungbean to characterize DNA variation patterns within and among closely related species (Dikshit et al., 2007; Raturi et al., 2012). DNA markers allow rapid identification of cultivars, hybrids, somaclonal variants, and clones with high efficiency and less labor cost (Reddy et al., 2002). In the present study, we successfully applied RAPD, URP, SSR, and SSR-RIS markers for molecular confirmation of 36 putative recombinants (Mungbean × Mashbean) that could not be discriminated from the self-pollinated progeny of the female parent on the basis of morphological characteristics.

## Materials and Methods

The present investigation was carried out at Plant Breeding and Genetics Division, Nuclear Institute for Agriculture and Biology, Faisalabad during the year, 2012–2013. The experimental material comprised of thirty six recombinant genotypes (**Table 1**) selected from field experiment along with three mungbean parents (Var.6601, NM-92, and NM-2006) and three mashbean parents (Mash-97, Mash-88, and Mash 3-156-1). Recombinant genotypes were evaluated at molecular level to confirm the introgression of mashbean genome into mungbean background. Seedlings of parental genotypes and recombinants were grown in petri plates at room temperature and young leaf tissues were used for extraction of DNA using the method described by Plaschke et al. (1995).

**Table 1:** Interspecific recombinant genotypes used for DNA Analysis.

Sr.#	Genotype	Parentage	Sr.#	Genotype	Parentage
1	MMH 11534	Var.6601 × Mash 3-156-1	19	MMH 9125	Var.6601 × Mash 3-156-1
2	MMH 1125	NM-92 × Mash-97	20	MMH 13115	Var.6601 × Mash 3-156-1
3	MMH 4615	NM-92 × Mash-97	21	MMH 16111	Var.6601 × Mash 3-156-1
4	MMH 53105	NM-92 × Mash-97	22	MMH 16425	Var.6601 × Mash 3-156-1
5	MMH 5615	NM-92 × Mash-97	23	MMH 24425	Var.6601 × Mash 3-156-1
6	MMH 2133	NM 2006 × Mash 88	24	MMH 37414	Var.6601 × Mash 3-156-1
7	MMH 2225	NM 2006 × Mash 88	25	MMH 210115	NM-92 × Mash-97
8	MMH 4255	NM 2006 × Mash 88	26	MMH 3132	NM-92 × Mash-97
9	MMH 7112	Var.6601 × Mash 3-156-1	27	MMH 4335	NM-92 × Mash-97
10	MMH 1312	NM-92 × Mash-97	28	MMH 2131	NM 2006 × Mash 88
11	MMH 3563	NM-92 × Mash-97	29	MMH 2333	NM 2006 × Mash 88
12	MMH 3615	NM-92 × Mash-97	30	MMH 4211	NM 2006 × Mash 88
13	MMH 1115	NM 2006 × Mash 88	31	MMH 4224	NM 2006 × Mash 88
14	MMH 2112	NM 2006 × Mash 88	32	MMH 6235	NM 2006 × Mash 88
15	MMH 2121	NM 2006 × Mash 88	33	MMH 7124	NM 2006 × Mash 88
16	MMH 4295	NM 2006 × Mash 88	34	MMH 10212	Var.6601 × Mash 3-156-1
17	MMH 7111	NM 2006 × Mash 88	35	MMH 15135	Var.6601 × Mash 3-156-1
18	MMH 7142	NM 2006 × Mash 88	36	MMH 15334	Var.6601 × Mash 3-156-1

PCR was performed for RAPD (10), URP (12), and SSR (13) as described by Dikshit et al. (2007) and for SSR (RIS) (02) markers (**Table 2**). PCR mixtures were prepared containing 1X PCR buffer, 0.2 mM dNTPs, 1.5 mM MgCl_2_, 1 U Taq polymerase (Enzynomics 2X TOPsimple^TM^ DyeMIX-nTaq), 0.4 μM primer, and 100 ng template DNA. PCR amplifications for URP and SSR (RIS) markers were performed using thermal cycler (Bio RAD T-100, USA) with initial denaturation at 94°C for 3 min and then 40 cycles of denaturation at 94°C for 1 min, primer annealing at 55°C for 1 min, primer extension at 72°C for 2 min with a final extension step at 72°C for 10 min. PCR amplifications for RAPD were performed with initial denaturation at 94°C for 4 min and then 40 cycles of denaturation at 94°C for 30 s, primer annealing at 35°C for 1 min, primer extension at 72°C for 2 min and final extension step at 72°C for 7 min. PCR conditions used for SSR markers include initial denaturation at 94°C for 2 min and then 35 cycles of denaturation at 94°C for 30 s, primer annealing at 50–60°C for 30 s, primer extension at 72°C for 1 min and final extension step at 72°C for 10 min.

**Table 2:** Details of polymorphic RAPD, RIS, and SSR primers used for molecular confirmation of Mungbean × Mashbean interspecific recombinants.

Primer Code	Sequence (5′–3′)	Annealing temperature	Product size (bp)
**Random amplified polymorphic DNA (RAPD)**			
OPU-3	CTATGCCGA	35°C	397–2060
OPAJ-20	ACACGTGGTC	35°C	440–1870
OPS-07	TCCGATGCTG	35°C	420–1940
**SSR (RIS) primers**			
RIS-F	TAATTTCTGCTTGCTCCATGC	55° C	604–2260
RIS-R	ACTGGGGTGCACTGGATTAG	55°C	177–1828
**Simple sequence repeats (SSR)**			
VR040	(F) TGACAACATGGGAAGAAGAAGA (R) ACACCAACACAAAAGCAAACAC	52° C	157–197
VR062	(F) CGAAGACGAAATCTGAAGACAA (R) TTACTTCTCCCAGCACTCCAAT	52° C	138–156
VR0111	(F) TGCATCTTTATTGAGTTCCGTG (R) GTTTTGGGGTGAATGTTGGATA	55° C	190–222
VR0304	(F) GAAGCGAAGAAGCCATAGAAAA (R) CCTCACACACAACACAACAGAA	52° C	180–190

*(F) Forward, (R) Reverse*.

The amplified products for URP, RAPD, and SSR (RIS) were separated electrophoretically on 1.5% agarose gel in 1× TBE buffer along with molecular weight markers (Bio Basic Inc. M109-A/M109-B and Thermo Scientific SM0373), stained with ethidium bromide and visualized and photographed over a UV *trans* illuminator (UVP Photo Doc-It^TM^ Imaging System). The amplified products for SSR were separated electrophoretically (High throughput gel electrophoresis system, CBS Scientific) on 7% polyacrylamide gel, stained with ethidium bromide and visualized and photographed over a UV *trans* illuminator (UVP Photo Doc-It^TM^ Imaging System). Banding patterns were analyzed by using UVI-BandMap software.

## Results

**FIGURE 1:**
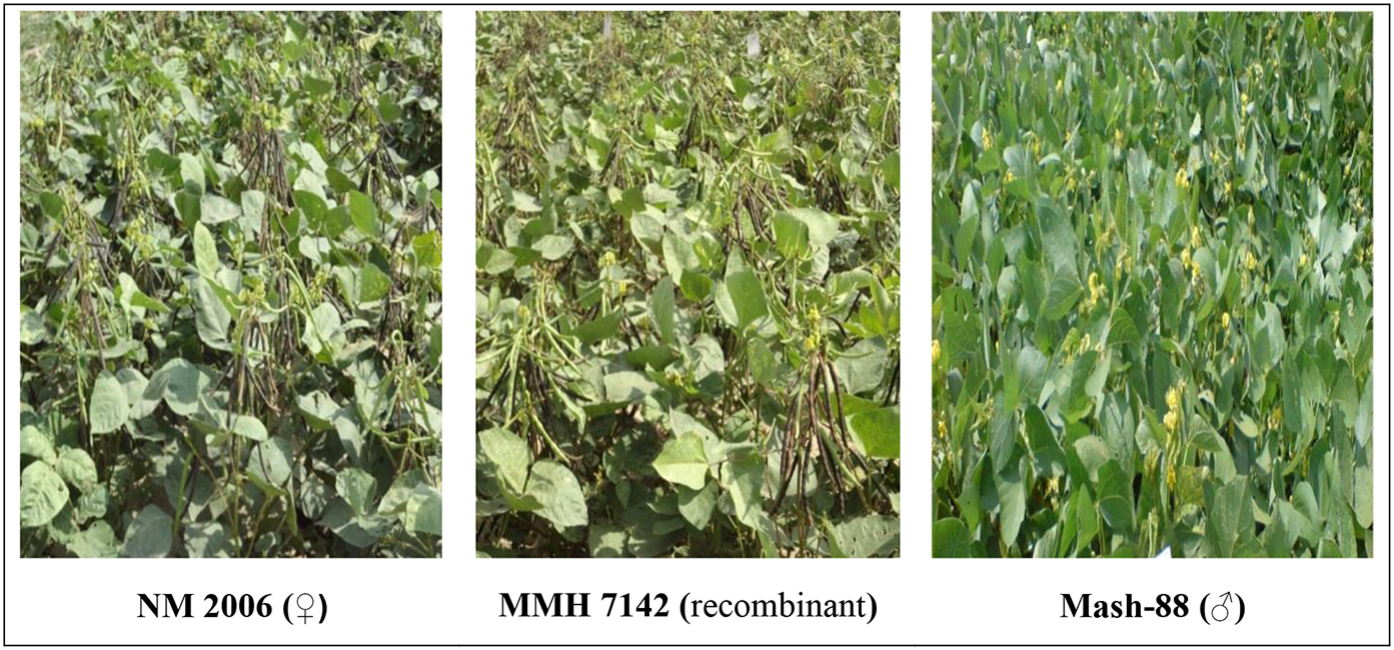
**Pictorial view of mungbean (female parent), mung × mash (recombinant) and mashbean (male parent)**.

Mungbean × mashbean interspecific recombinants that showed morphological characteristics similar to their respective female parents were verified as true recombinants by using RAPD, URP, and SSR markers. One example of the similarity of recombinants morphological characteristics with the female parent is given in **Figure 1**. Firstly male and female parents of the recombinants were screened to identify the genetic variation present among parent genotypes and then the screening of interspecific recombinants was performed.

### Parental Screening

**FIGURE 2:**
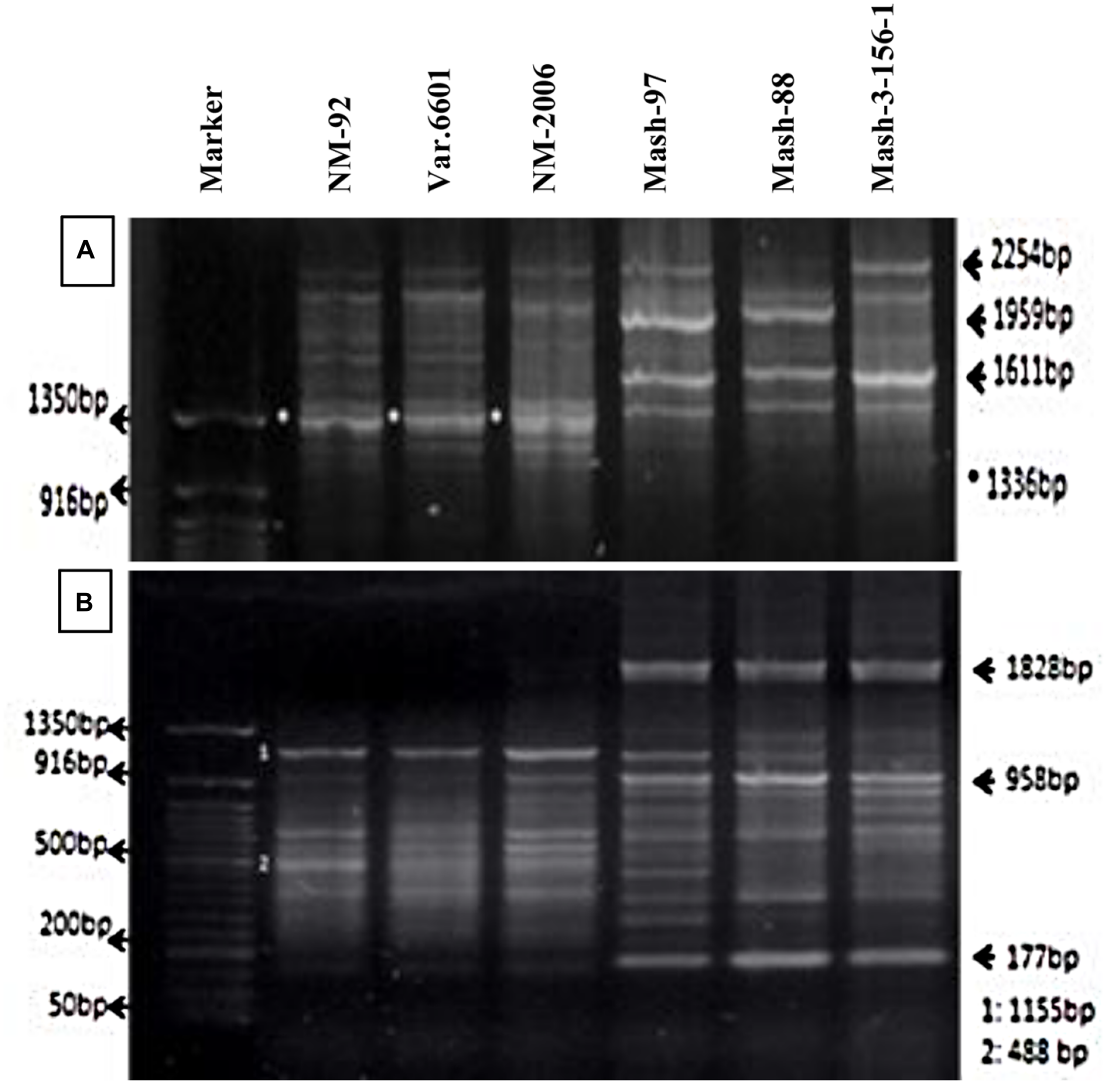
**PCR profiles of parental genotypes using RIS-F **(A)** and RIS-R **(B)** marker**.

Six parents (NM-92, Var.6601, NM-2006, Mash-97, Mash-88, and Mash-3-156-1) involved in crossing were tested for genetic variation present at DNA level. PCR profiling using RIS-F clearly differentiated mungbean and mashbean parents from each other and also showed polymorphism within the male and female parent varieties (**Figure 2A**). RIS-F amplified band size of 1336 bp for female parents and 1611 to 2254 bp for male parents. Mashbean genotypes, used as male parents, exhibited male parent specific marker which interestingly, also produced polymorphic banding pattern for all the mashbean genotypes. At least two genotype specific markers were differentiated in different mashbean genotypes. RIS-R clearly differentiated mungbean and mashbean parents from each other (**Figure 2B**). RIS-R amplified band size of 488 and 1155 bp for female parents (Mungbean) and 177, 958, and 1828 bp for male parent (Mashbean) genotypes involved in interspecific hybridization. The marker clearly differentiated not only mungbean and mashbean varieties from one another but intra varietal difference in mungbean and mashbean were also identified.

**FIGURE 3:**
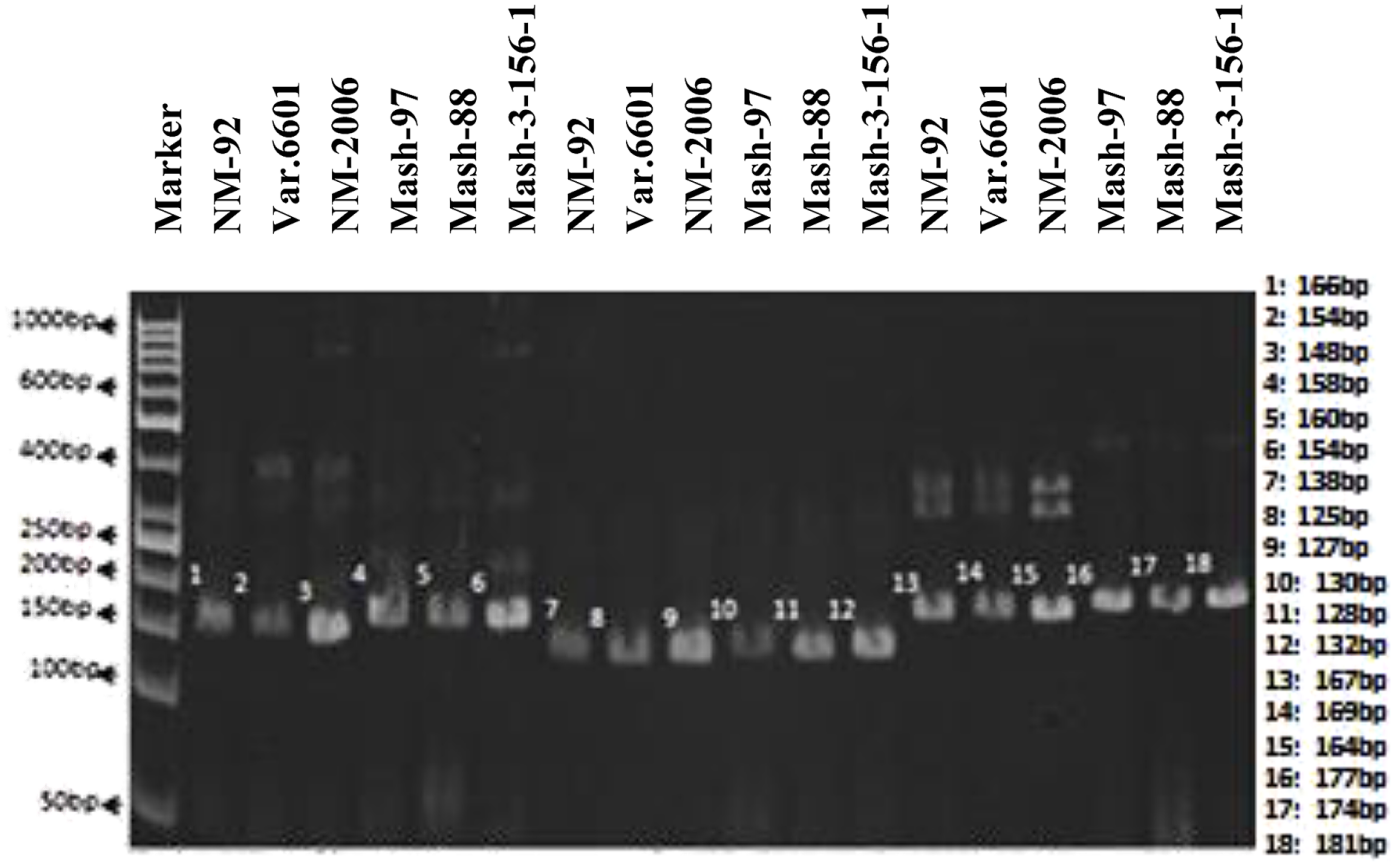
**PCR profiles of parental genotypes using SSR markers VR040 (L1–L6), VR062 (L7–L12) and VR0111 (L13–L18). ^∗^Marker for recombination**.

In case of SSR markers, out of 13 primers, only 3 (VR040, VR062, and VR0111) detected polymorphism between and among female and male parents. Thus, they proved to be an excellent resource for the identification of true recombinants (**Figure 3**). VR040 amplified bands of 148, 154, 166 bp for female parents and 154, 158, 160 bp for male parents. VR062 amplified bands of 125, 127, 138 bp for female parents and 128, 130, 132 bp for male parents. Similarly, VR0111 amplified bands of 164, 167, 169 bp for female parents and 174, 177, 181 bp for male parents.

**FIGURE 4:**
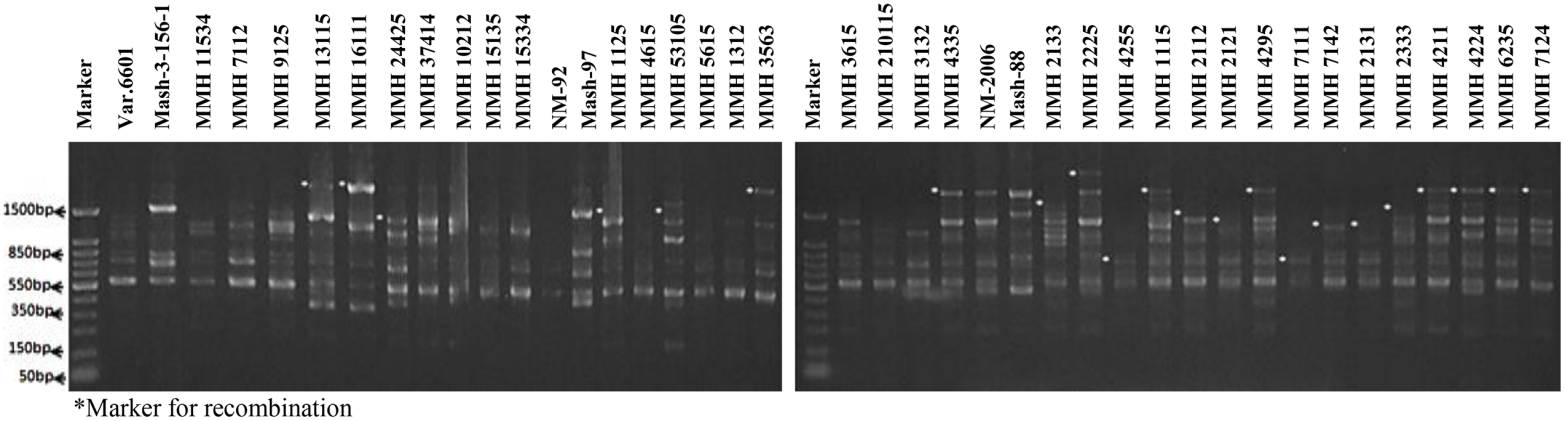
**PCR profiles of parental genotypes along with interspecific recombinants using RAPD primer OPU-3. ^∗^Marker for recombination**.

**FIGURE 5:**
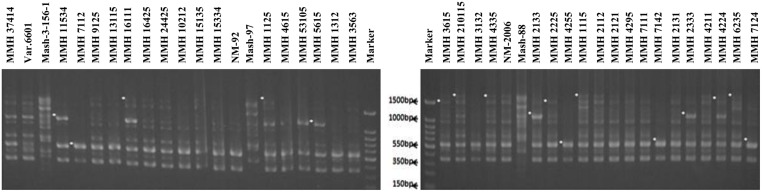
**PCR profiles of parental genotypes along with interspecific recombinants using RAPD primer OPAJ-20. ^∗^Marker for recombination**.

**FIGURE 6:**
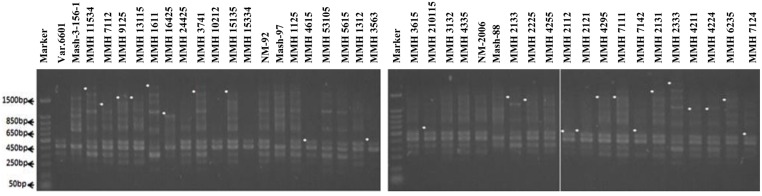
**PCR profiles of parental genotypes along with interspecific recombinants using RAPD primer OPS-07. ^∗^Marker for recombination**.

Molecular screening of parent genotypes was also performed along with interspecific recombinants through RAPD markers. Primer OPU-3 showed polymorphic results and clearly differentiated all parental cross combinations (**Figure 4**). In cross combination Var.6601 × Mash-3-156-1, female parent was clearly different from male parent. Similarly, polymorphic banding pattern was observed among other cross combinations which showed clear genetic differentiation between female and male parents. Primer OPU-3 also showed polymorphic banding patterns among female and male parent genotypes. Primer OPAJ-20 successfully differentiated female and male parents (**Figure 5**) and also showed genetic variability among mungbean and mashbean genotypes. Due to its highly polymorphic nature, it can be used for genetic differentiation of interspecific recombinant genotypes from their parents. Another RAPD marker, OPS-7 showed clear differences between male and female parents of all cross combinations. It showed polymorphism among male and female parent genotypes (**Figure 6**) and proved to be efficient marker for screening true recombinants.

In general, all polymorphic RIS (RIS-F and RIS-R), SSR (VR040, VR062, and VR0111) and RAPD markers (OPU-3, OPAJ-20, and OPS-07) showed genetic differentiation not only between male and female genotypes of all cross combinations but also among mungbean (female parent) and mashbean (male parent) varieties/genotypes. These markers were found to be efficient for the identification of interspecific recombinants.

### Confirmation of Interspecific Recombinants

PCR profile of parents and recombinants of Mungbean × Mashbean crosses by using RAPD marker OPU-3 is presented in **Figure 4**. OPU-3 showed polymorphic banding pattern when tested on recombinants along with their parents. This primer amplified different band sizes ranging from 397 to 2060 bp. OPU-3 confirmed 22 recombinants out of which 5 recombinants, i.e., MMH 4295, MMH 4211, MMH 4224, MMH 6235, and MMH 7124 showed clear male parent specific band. While remaining 17 recombinants were confirmed through diversified PCR profiles that were different from male and female parent (**Table 3**). Another RAPD primer OPAJ-20 which found to be polymorphic in parental screening and showed clear genetic differentiation between male and female parents as well as among male and female parent genotypes was used to confirm interspecific recombinants. OPAJ-20 produced multiple bands ranging from 440 to 1870 bp (**Figure 5**) and confirmed 17 interspecific recombinants (**Table 3**). Out of these 17 recombinants, 6 (MMH 2225, MMH 3615, MMH 1115, MMH 210115, MMH 4335, and MMH 6235) were confirmed through male parent specific bands. The remaining 11 recombinants were confirmed through diversified PCR profiles as compared to respective parents.

**Table 3:** Confirmation of inter-specific Mung × Mash recombinant genotypes through RIS and RAPD analysis.

Recombinant Genotypes	Molecular markers			


	RIS-F	RAPD-OPU-3	RAPD-OPAJ-20	RAPD-OPS-07
MMH 11534	R	–	R	♂
MMH 1125	♂	R	♂	–
MMH 4615	–	–	–	R
MMH 53105	R	R	–	–
MMH 5615	R	–	R	–
MMH 2133	–	R	R	♂
MMH 2225	♂	R	♂	♂
MMH 4255	♂	R	R	–
MMH 7112	R	–	R	♂
MMH 1312	–	–	–	–
MMH 3563	–	R	–	R
MMH 3615	–	–	♂	–
MMH 1115	♂	R	♂	–
MMH 2112	♂	R	–	R
MMH 2121	R	R	–	R
MMH 4295	♂	♂	–	♂
MMH 7111	♂	R	–	♂
MMH 7142	♂	R	R	R
MMH 9125	–	–	–	♂
MMH 13115	–	R	–	♂
MMH 16111	♂	R	R	♂
MMH 16425	♂	–	–	R
MMH 24425	–	R	–	–
MMH 37414	–	–	–	♂
MMH 210115	♂	–	♂	R
MMH 3132	–	–	–	–
MMH 4335	–	R	♂	–
MMH 2131	–	R	–	♂
MMH 2333	♂	R	R	♂
MMH 4211	♂	♂	–	♂
MMH 4224	♂	♂	R	♂
MMH 6235	♂	♂	♂	♂
MMH 7124	♂	♂	R	♂
MMH 10212	R	–	–	–
MMH 15135	–	–	–	♂
MMH 15334	R	–	–	–

*Male-specific band; –, Not confirmed; R, Recombinant*.

Among RAPDs, OPS-07 confirmed maximum number of recombinants with band sizes ranging from 420 to 1940 bp. This primer showed clear genetic differences between female and male parent genotypes. It also showed polymorphism among mungbean (female parent) and mash bean (male parent) genotypes (**Figure 6**). A total of 24 genotypes out of 36 were confirmed as recombinant genotypes (**Table 3**). Clear male parent specific markers were detected in 17 recombinants by using this primer.

**FIGURE 7:**
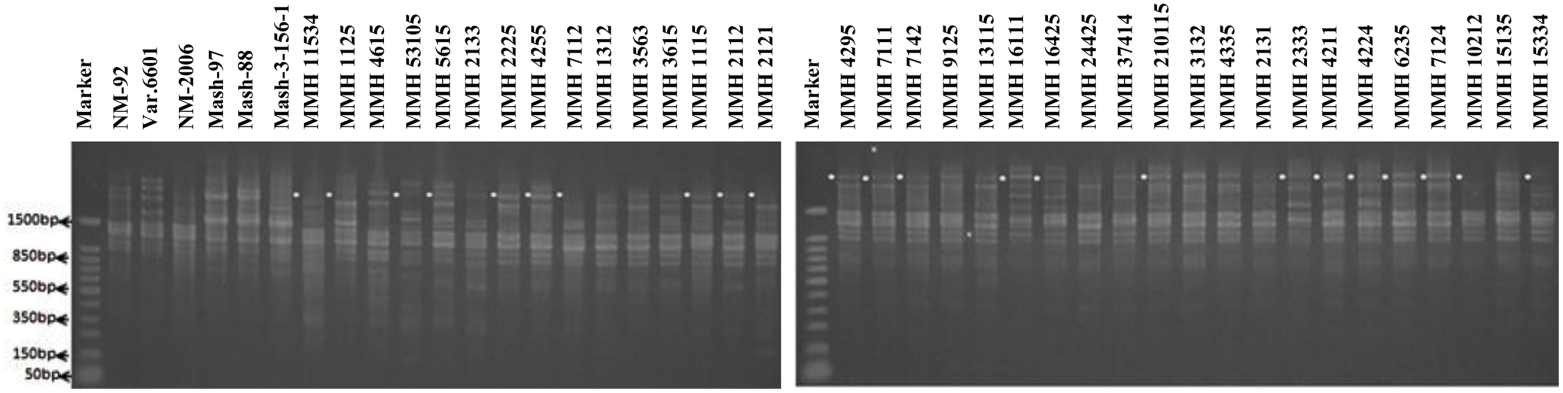
**PCR profiles of parental genotypes along with interspecific recombinants using RIS-F. ^∗^Marker for recombination**.

Among SSR (RIS) primers, the polymorphic banding pattern of RIS-F when tested on different recombinants along with their parents (**Figure 7**) confirmed 23 out of 36 recombinants. Clear male parent specific bands were detected in 16 recombinants by using this marker. Moreover, some recombinants were confirmed through diversified PCR profiles as compared to male and female parent (**Table 3**). This primer amplified different band sizes ranging from 604 to 2260 bp.

**FIGURE 8:**
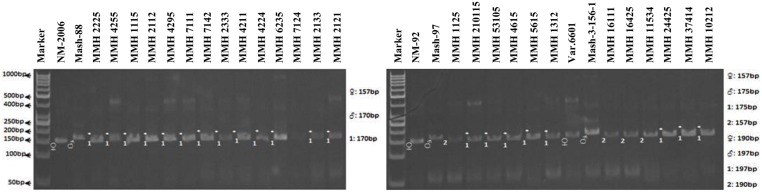
**PCR profiles of parental genotypes along with interspecific recombinants using SSR marker VR040. ^∗^Marker for recombination**.

**FIGURE 9:**
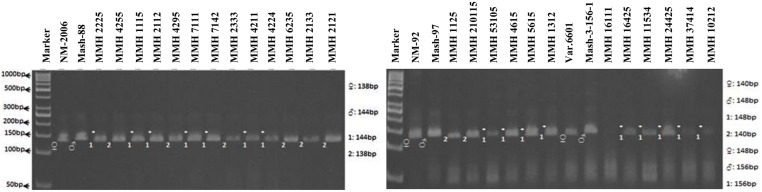
**PCR profiles of parental genotypes along with interspecific recombinants using SSR marker VR062. ^∗^Marker for recombination**.

Simple sequence repeat markers further confirmed recombinations in recombinants selected from RAPD and RIS analysis. For this purpose a total of 13 SSR markers were used and only four were found polymorphic, whereas others showed monomorphic banding patterns. VR040 was one of the SSR markers which clearly differentiated male and female parent genotypes and showed polymorphic banding patterns in recombinants analyzed in this study. Distinct male parent specific markers were detected by using this primer (**Figure 8**). This primer amplified band sizes of 157 bp for female parent and 170 bp for male parent in NM-2006 × Mash-88 cross combination and amplified a band of 170 bp in recombinants. VR040 amplified band sizes of 157 and 175 bp for female and male parents respectively for NM-92 × Mash-97 cross combination. For Var.6601 × Mash-3-156-1 it produced band size of 190 bp for female parent and 197 bp for male parent. VR040 showed male specific bands in 21 out of 26 recombinants. The remaining five recombinants (MMH 7124, MMH 1125, MMH 1611, MMH 16425, and MMH 11534) showed female specific marker, hence not confirmed as true recombinants by using this primer (**Table 4**). Another SSR marker, VR062 which showed polymorphic banding pattern in parental genotypes also revealed clear differentiation in recombinants in comparison with their respective female and male parent genotypes. Band sizes of male specific amplification products, i.e., 144, 148, and 156 bp were found in NM-2006 × Mash-88, NM-92 × Mash-97, and Var.6601 × Mash-3-156-1 recombinants, respectively. This primer declared 17 recombinants true recombinants since they showed male parent specific banding pattern (**Table 4**). The remaining nine recombinants were not confirmed by this marker as they showed their respective female parent specific banding pattern. Many of the recombinants identified by VR062 were also indicated as true recombinants by VR040. The PCR profiles of recombinants along with their parents amplified by VR062 are presented in **Figure 9**.

**Table 4:** Re-confirmation of inter-specific Mung × Mash recombinant genotypes through SSR analysis.

Recombinant Genotypes	SSR markers			
	VR040	VR062	VR0111	VR0304
MMH 16425	–	R	R	R
MMH 2112	R	R	R	R
MMH 16111	–	–	R	R
MMH 7111	R	R	R	R
MMH 7142	R	R	R	R
MMH 6235	R	–	R	R
MMH 4255	R	–	R	R
MMH 1115	R	R	R	R
MMH 4295	R	–	R	R
MMH 4224	R	R	R	–
MMH 7124	–	R	R	–
MMH 2333	R	–	R	–
MMH 1125	–	–	R	R
MMH 4211	R	R	R	R
MMH 2225	R	R	R	R
MMH 210115	R	–	R	R

*–, Not confirmed; R, Recombinant*.

**FIGURE 10:**
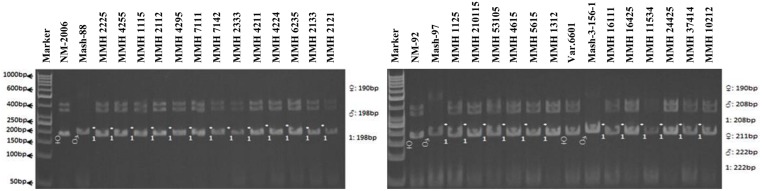
**PCR profiles of parental genotypes along with interspecific recombinants using SSR marker VR0111. ^∗^Marker for recombination**.

**FIGURE 11:**
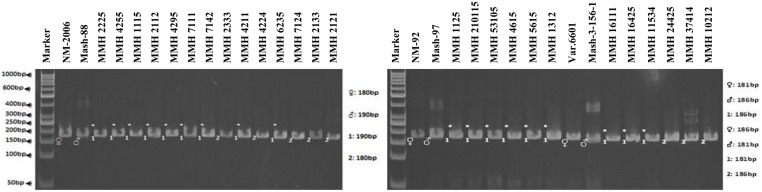
**PCR profiles of parental genotypes along with interspecific recombinants using SSR marker VR0304. ^∗^Marker for recombination**.

Simple sequence repeat marker VR0111 was efficient in detecting genetic variability between female and male parent genotypes. In female parent genotypes, this primer amplified band sizes of 190 bp in NM-2006 and NM-92 and 211 bp in Var.6601 whereas in male parent genotypes it amplified 198 bp in Mash-88, 208 bp in Mash-97, and 222 bp in Mash-3-156-1. This primer amplified male specific band sizes of 198, 208, and 222 bp in the recombinants (**Figure 10**). This was the only primer which showed complete male parent specific banding pattern thus declaring maximum number of recombinants as true recombinants (**Table 4**). Distinct genetic differentiation of parental genotypes was also revealed by SSR marker VR0304. This primer amplified band sizes of 180 (NM-2006), 181 (NM-92), 186 bp (Var.6601) in female parents and 181 (Mash-3-156-1), 186 (Mash-97), 190 bp (Mash-88) in male parents while male specific band sizes of 181, 186, and 190 bp in recombinants. The PCR profiles of recombinants along with their respective female and male parent genotypes (**Figure 11**) confirmed 18 recombinants out of 26 genotypes (**Table 4**). MMH 2333, MMH 4224, MMH 7124, MMH 2133, MMH 2121, MMH 24425, MMH 37414, and MMH 10212 showed female specific marker, hence not confirmed as true recombinants by using this primer. VR040 also did not confirmed MMH 7124 as recombinants. The recombinants, MMH 2333, MMH 2133, and MMH 2121 were also not confirmed by VR 062.

## Discussion

Our investigation showed RAPD, URP, and SSR markers to be efficient tools in discriminating the interspecific recombinants from the self-pollinated progeny of female parents. These markers can be effectively used to fingerprint and differentiate plants with highly similar morphological characteristics. The recombination status of hybrids can be confirmed by comparing the amplified polymorphic bands between recombinants and female parents (male parent specific bands). RAPD (OPU-3, OPAJ-20, and OPS-07) depicted polymorphism between male and female parents and among recombinants. Many other studies reported that RAPD markers are efficient in amplifying DNA from dispersed polymorphic loci from the genome (Baral and Bosland, 2002; Rasul et al., 2007; Wang et al., 2011). These markers can be used to assess genetic variability among and between *Vigna* species (Kaga et al., 1996; Santalla et al., 1998; Lakhanpaul et al., 2000; Ba et al., 2004; Saini et al., 2004; Raturi et al., 2012). Wang et al. (2008) reported thirty gene derived markers which were employed to reveal interspecific phylogenetic relationships and genetic diversity among 48 accessions of 12 *Vigna* species. Elham et al. (2010) used RAPD markers for determining genetic relationship among *Vigna* species. Souframanien and Gopalakrishna (2004) studied the DNA polymorphism in 18 elite blackgram genotypes by using RAPD and ISSR markers. Amplification of genomic DNA of blackgram genotypes, using RAPD analysis, yielded 44 polymorphic fragments varied in size from 200 bp (OPA-13) to 2500 bp (OPK-4). Similarly, Srivastava et al. (2011) used RAPD markers for genetic diversity analysis of blackgram genotypes. Total amplified fragments were 346 out of which 338 were polymorphic (97.68%) with fragment size varied from 50 to 3000 bp.

Similarly, 12 URP markers were used out of which some markers did not show any amplification and some showed very little or no polymorphism. After testing RAPD and URP markers, the recombinants were screened with 2 SSR (RIS) primers and both primers showed polymorphic banding pattern. RIS primers separately and at lower annealing temperature amplified products in mung × mash recombinants and may be used as potential markers. Thereafter, 13 SSR markers were used out of which only four markers, VR040, VR062, VR0111, and VR0304 exhibited polymorphism. Dikshit et al. (2007) also used URP, RAPD, and SSR markers for genetic differentiation of *Vigna* species and concluded that SSR marker system was more efficient in detecting genetic variability among all the *Vigna* species. Dikshit et al. (2012) used 78 SSR’s, 41 were amplified in one *Vigna* species and out of this 36 were observed to be polymorphic. Kajonphol et al. (2012), found 21 SSR markers to be common between mashbean and mungbean. Gupta and Varshney (2000) and Hernandez et al. (2002) used SSR markers very effectively in marker assisted selection, genotyping, and gene mapping. Chen et al. (2015) identified EST-SSR markers through transcriptome sequencing of mungbean genes. Out of 200 randomly selected SSR loci, 66 primer pairs produced reproducible amplicons that were polymorphic among 31 mungbean accessions. Wang et al. (2015) carried out SSR analysis of mungbean based on an SSR enriched library and reported 387 validated and mapped markers that can be used in marker assisted selection in mungbean.

## Conclusion

All the three maker systems used showed polymorphism between mashbean (male) and mungbean (female) and among male and female parent genotypes except URP. They revealed the presence of genetic variation in the investigated material that can be exploited for an efficient breeding program. Among various crosses, NM 2006 × Mash 88 was found to be the most successful interspecific cross as highest proportion of the genetically confirmed recombinants belonged to this cross combination. Comparison of marker systems confirmed the SSR efficiency in discerning genetic variability and recombination with reference to specific chromosome number and loci. RIS and RAPD were found to be more efficient in amplifying DNA from dispersed polymorphic loci from the genome and may be used for recombinant identification where SSR fails to detect polymorphism. In conclusion, marker assisted selection approach is helpful in selecting true interspecific recombinants among Mungbean × Mashbean. This approach can differentiate the interspecific recombinant seed from self-pollinated seed; thus can overcome the issues related to environment and inadequacy of morphological characteristics alone.

## Author Contributions

GA, NI, AH, MJA and MA were involved in planning of the study. MR and AH were involved in execution of practical lab work and data analysis. AH, MR, GA and NI contributed in manuscript writing and finalization of draft.

## Conflict of Interest Statement

The authors declare that the research was conducted in the absence of any commercial or financial relationships that could be construed as a potential conflict of interest.
